# Towards personalised allele-specific CRISPR gene editing to treat autosomal dominant disorders

**DOI:** 10.1038/s41598-017-16279-4

**Published:** 2017-11-23

**Authors:** Kathleen A. Christie, David G. Courtney, Larry A. DeDionisio, Connie Chao Shern, Shyamasree De Majumdar, Laura C. Mairs, M. Andrew Nesbit, C. B. Tara Moore

**Affiliations:** 1 Biomedical Sciences Research Institute, Ulster University, Coleraine, Northern Ireland BT52 1SA UK; 2 Avellino Laboratories, Menlo Park, California, CA 94025 USA

## Abstract

CRISPR/Cas9 holds immense potential to treat a range of genetic disorders. Allele-specific gene disruption induced by non-homologous end-joining (NHEJ) DNA repair offers a potential treatment option for autosomal dominant disease. Here, we successfully delivered a plasmid encoding *S. pyogenes* Cas9 and sgRNA to the corneal epithelium by intrastromal injection and acheived long-term knockdown of a corneal epithelial reporter gene, demonstrating gene disruption via NHEJ *in vivo*. In addition, we used *TGFBI* corneal dystrophies as a model of autosomal dominant disease to assess the use of CRISPR/Cas9 in two allele-specific systems, comparing cleavage using a SNP-derived PAM to a guide specific approach. *In vitro*, cleavage via a SNP-derived PAM was found to confer stringent allele-specific cleavage, while a guide-specific approach lacked the ability to distinguish between the wild-type and mutant alleles. The failings of the guide-specific approach highlights the necessity for meticulous guide design and assessment, as various degrees of allele-specificity are achieved depending on the guide sequence employed. A major concern for the use of CRISPR/Cas9 is its tendency to cleave DNA non-specifically at “off-target” sites. Confirmation that *S. pyogenes* Cas9 lacks the specificity to discriminate between alleles differing by a single base-pair regardless of the position in the guide is demonstrated.

## Introduction

**Figure 1:**
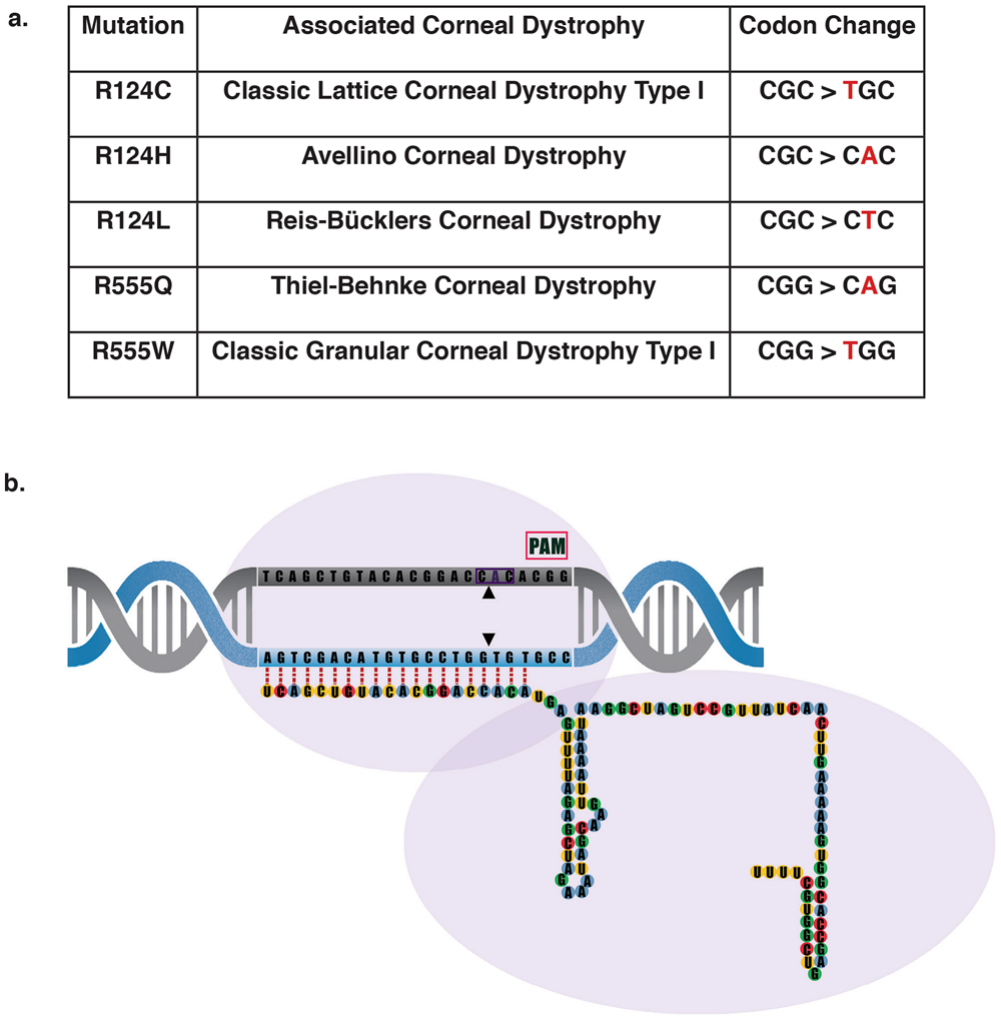
*S. pyogenes* Cas9 to treat dominant negative *TGFBI* corneal dystrophies. (**a**) Cas9 (purple outline) can be directed to cut any sequence in the genome (DNA target in grey), provided it is directly upstream of a protospacer adjacent motif known as PAM (pink box). This can be achieved by altering the 20 nucleotide guide sequence, which is associated with a 82 nucleotide scaffold. (**b**) 5 prevalent *TGFBI* mutations and their associated corneal dystrophy and codon change. (**c**) Schematic of the position of the 60 missense mutations across the *TGFBI* gene. The hotspots at exons 4, 11, 12 and 14 are evident, with exons 4 and 12 expanded to show the location of the 5 most prevalent *TGFBI* mutations; R124C, R124H, R125L, R555Q and R555W.

The promise of personalised gene therapy has been brought nearer fruition with the recent advances in the field of genome engineering, particularly the development of Clustered Regularly Interspaced Palindromic Repeats (CRISPR)/CRISPR associated protein (Cas) systems. CRISPR/Cas9 is an RNA guided endonuclease, that has been manipulated for use in mammalian cells to act as a two component system, requiring only a Cas9 nuclease and a single guide RNA (sgRNA)^1–3^. Cas9 can be directed to cut a desired sequence in the genome, provided it is directly upstream of a protospacer adjacent motif (PAM), by simply altering the guide RNA sequence (Fig. 1a). The site-specific sgRNA will direct the Cas9 nuclease to make a double strand break (DSB). The cell will then attempt to repair this damage, by either error-prone non-homologous end joining (NHEJ) or precise homology directed repair (HDR)^4^, and it is by these different cellular responses that different forms of gene editing can be achieved.

NHEJ can be utilised to generate gene knockouts, due to the high frequency of frameshifting mutations generated^4,5^. Allele-specific gene disruption via NHEJ is a potential approach to treat dominant negative disorders, in which the causative gene is haplosufficient; this involves targeting the mutant allele alone for disruption, leaving the wild-type allele intact and restoring the phenotype^6–9^. This approach relies on the ability of the targeting system to unequivocally discriminate between wild-type and mutant sequence.

Although CRISPR/Cas9 holds immense promise, one caveat to the use of the system is that Cas9 nuclease has been shown to tolerate mismatches between the guide sequence and the target^10,11^. This can lead to off-targeting elsewhere in the genome or, indeed in this case, cleavage of the wild-type allele. Efforts have been made to increase the specificity of Cas9 and eliminate off-target cutting, including; use of truncated guides^12^, Cas9 variants from other bacterial species to exploit more intricate PAMs^13^, rationally engineering the Cas9 nuclease^14^, and using the mutant sequence to induce specificity such as utilising a novel SNP-derived PAM^15,16^.

In particular utilising a novel PAM is an attractive option for allele-specific editing. Previous work has demonstrated that when mutations result in a novel PAM, guide RNAs can be designed, utilising this new PAM, allowing only the mutant allele to be targeted, producing an allele-specific knockout^14^.

The cornea offers an ideal platform for testing personalised gene therapy, due to its immediate accessibility, small surface area and immune-privileged status. Collectively the corneal dystrophies represent a group of inheritable blinding diseases that alter the shape or transparency of the cornea. Currently mutations in 14 genes are associated with corneal dystrophies, 9 of them presenting with an autosomal dominant inheritance pattern^17^. Corneal dystrophies linked to these 9 genes predominantly result from missense mutations or small in-frame insertions or deletions that cause disease by a dominant negative effect of the mutant protein^17^.

Transforming growth factor beta induced protein (*TGFBI*p) has been linked to a range of stromal or stromal-epithelial corneal dystrophies^18–20^. *TGFBI* is predominantly produced in the corneal epithelium and is transported to the stromal layer, where the mutant protein accumulates^21^. To-date a total of 60 missense mutations in *TGFBI* have been linked to various corneal dystrophies^22,23^. These mutations span the entire *TGFBI* gene but are clustered in hotspots found in exon 4, 11, 12 and 14.

Despite the wide spectrum of mutations, the vast majority of cases are due to 5 prevalent mutations found in either codon 124 (exon 4) or codon 555 (exon 12)^24,25^ (Fig. 1b). These 5 mutations include; R124C, R124H, R124L, R555Q and R555W and as described account for the bulk of reported cases of *TGFBI* corneal dystrophies^26–28^. Remarkably, each of these mutations, differing by only a single amino acid, result in strikingly different protein aggregates with a very strong genotype-phenotype correlation.

To achieve complete allele-specificity for a particular *TGFBI* mutation, stringent fidelity is required as an almost perfect off-target site exists in the form of the wild-type allele, which, for the majority of *TGFBI* mutations, differs by only one base pair from the mutant. This report uses TGFBI corneal dystrophies as a model of autosomal dominant disease to assess the specificity of the CRISPR/Cas9 system for autosomal dominant disorders. An allele-specific approach to target the five most prevalent *TGFBI* corneal dystrophy mutations is investigated, which highlights the promiscuity of Cas9 and the need for a validated, highly specific approach, that will encompass all possible *TGFBI* mutations.

## Results

### *In vivo* corneal gene disruption induced by CRISPR/Cas9 gene editing and NHEJ-mediated DNA repair

**Figure 2:**
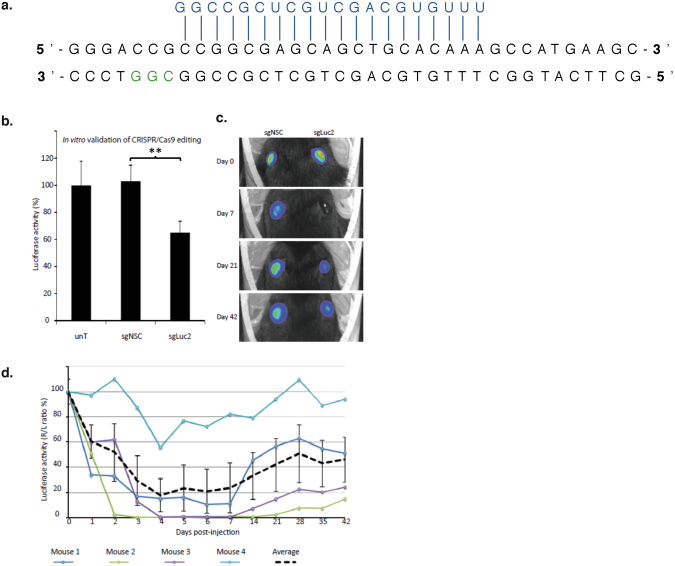
Sustained CRISPR/Cas9 mediated silencing of *luc2 in vivo*. (**a**) The short guide RNA (sgRNA) specific for *luc2* was designed to target the 5′ region of the gene, to increase the likelihood of inducing a frame-shifting deletion that would knock out luciferase activity by generating a premature termination codon. (**b**) An *in vitro* dual-luciferase assay demonstrated successful targeting of *luc2* by the sgLuc2 construct, as shown by a significant reduction in luciferase activity when normalized to untreated cells (data normalised against the untreated control = 100%). (**c**) Representative image of mice displaying a maximal reduction in *luc2* expression after injection with the sgLuc2 construct (right eye). This image was taken from the mouse represented by the green line in panel (d), below, at 7 days post treatment. (**d**) After treatment, the corneal luciferase activity of each mouse was quantified using a Xenogen IVIS live animal imager every day for 7 days, then every 7 days thereafter, for a total of 6 weeks. Luciferase activity for each treatment group expressed as a percentage of control (R/L ratio %).

We utilised a previously reported reporter knock-in mouse (*Krt12*+*/luc2*), that exclusively expresses firefly luciferase (luc2) in the corneal epithelium under control of the keratin K12 promoter, to study corneal delivery and activity of CRISPR/Cas9-mediated gene editing in living animals. To target the luciferase gene, an sgRNA utilising a PAM site 61 nucleotides downstream of the luc2 start codon was designed (Fig. 2a) and validated using a dual-luciferase assay (Fig. 2b). Therapeutic efficacy of this CRISPR/Cas9 system in living cornea was assessed following a single intrastromal injection in Krt12+/luc2 mice. Luciferase activity was evaluated with daily measurements up to 1 week following injection and weekly thereafter, for an additional 5 weeks (Fig. 2c). Luciferase activity was significantly reduced from post-injection day 1, with maximal silencing of >99% achieved at day 3 in one animal, and a maximal mean reduction of 82% ± 13% observed in 4 mice on day 4. Sustained silencing of luciferase expression was observed in 3 out of 4 mice over the entire monitoring period of the experiment (7 weeks), while in the remaining animal, luciferase inhibition persisted for 2 weeks (Fig. 2c).

### Mutational analysis of *TGFBI* corneal dystrophy mutations

Currently the best characterised CRISPR/Cas9 system is that of *Streptococcus pyogenes* (*Sp*Cas9), which recognises a 5′-NGG-3′ PAM. An analysis of the 60 known *TGFBI* missense mutations (See Supplementary Table 1) was performed to determine if i) they generate a novel *S. pyogenes* PAM or ii) they have a *S. pyogenes* PAM nearby, placing the mutation within the seed region, defined here as the first 8 nucleotides immediately adjacent to the PAM^29–31^.

**Figure 3:**
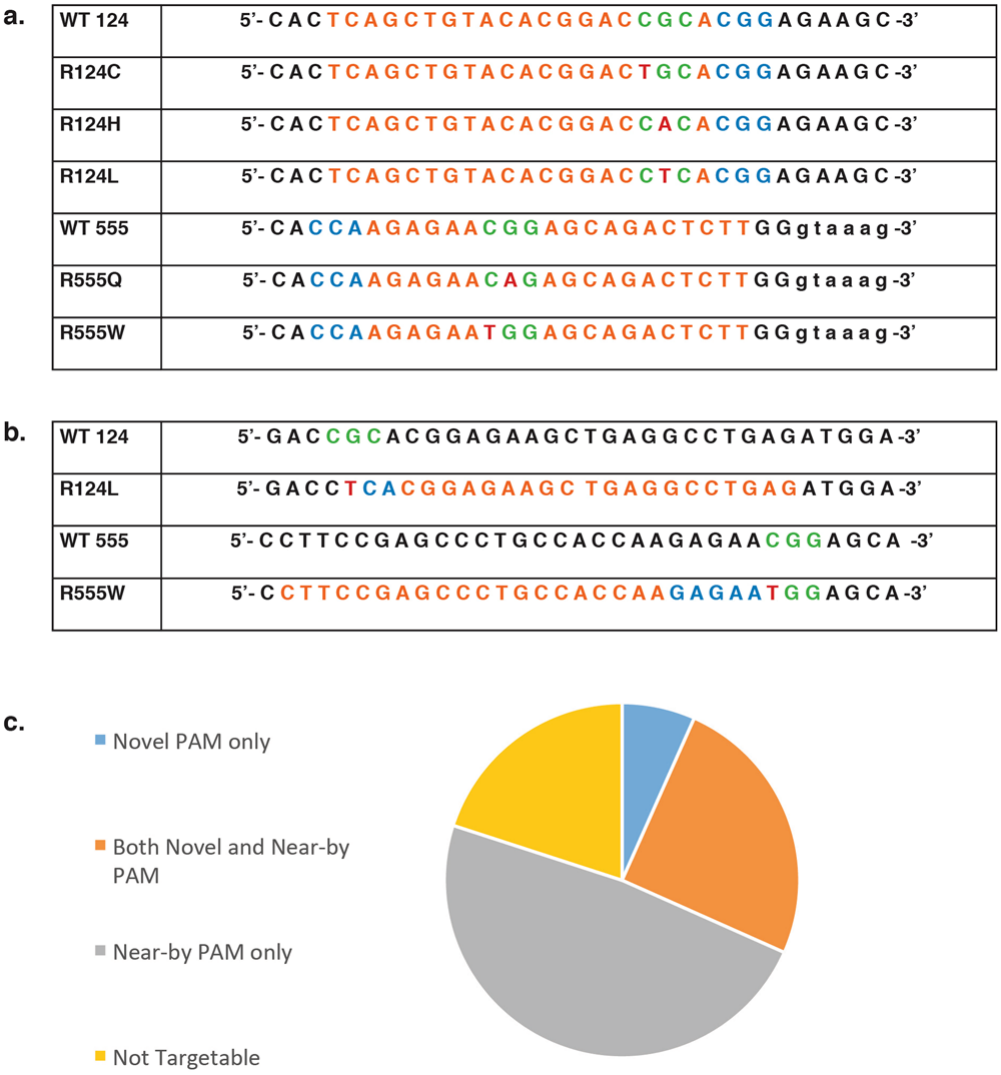
Analysis of *TGFBI* corneal dystrophy mutations in a CRISPR system Codons 124 or 555 shown in green, mutated base shown in red, nearest PAM to be utilised shown in blue and consequential guide sequence shown in orange. (**a**) Mutation analysis revealed that none of the prevalent *TGFBI* mutations generated a novel *S. pyogenes* PAM, however a naturally occurring PAM exists for all five mutations. For mutations in codon 124 the nearest downstream PAM places the mutated base at either position 3 or 4 of the guide sequence. For mutations in codon 555 the nearest downstream PAM places the mutated base at either position 7 or 8 of the guide sequence. (**b**) Mutational analysis revealed that R124L generates a novel PAM with a mutant *AsCpf1* that recognises a 5′-VYCV-3′ PAM. R124L generates a 5′-CTCA-3′ PAM. Further analysis revealed that R555W generates a novel PAM with *S.aureus* which is capable of recognising a 5′-NNGRRT-3′ PAM. R555W generates a 5′-GAGAAT-3′ PAM. (**c**) Venn diagram to illustrate the total number of *TGFBI* mutations that (i) generate a novel *S. pyogenes* PAM, (ii) have a near-by *S. pyogenes* PAM i.e. within the first 8 bp of the guide sequence, (iii) have both a novel and near-by *S. pyogenes* PAM or (iv) are not targetable by either approach.

19/60 mutations generate a novel *S. pyogenes* PAM, while 44/60 have a naturally occurring adjacent PAM site that places the mutation within an eight nucleotide seed region. When these figures are considered together, 20% of the *TGFBI* missense mutations are not targetable by *S. pyogenes* (Fig. 3a).

Analysis of the most prevalent *TGFBI* mutations in codon 124 and codon 555 revealed that none generated a novel *S. pyogenes* PAM, however all mutations had a *S. pyogenes* PAM within the first eight nucleotides of the target sequence (Fig. 3b).

Further to this, an analysis was conducted to determine if any of the prevalent *TGFBI* mutations generated a novel PAM with a CRISPR system from a different bacterial species. It was found that the R555W mutation generated a novel PAM with *Staphylococcus aureus* Cas9 (*Sa*Cas9), which recognises a 5′-NNGRRT-3′ PAM. In addition to this the R124L mutation generated a novel PAM with a mutant *Acidaminococcus* Cpf1 (*As*Cpf1) system^32^, which is capable of recognising a 5′-VYCV-3′ PAM (Fig. 3c).

### Validation of an *S. pyogenes* Cas9 PAM-specific approach

**Figure 4:**
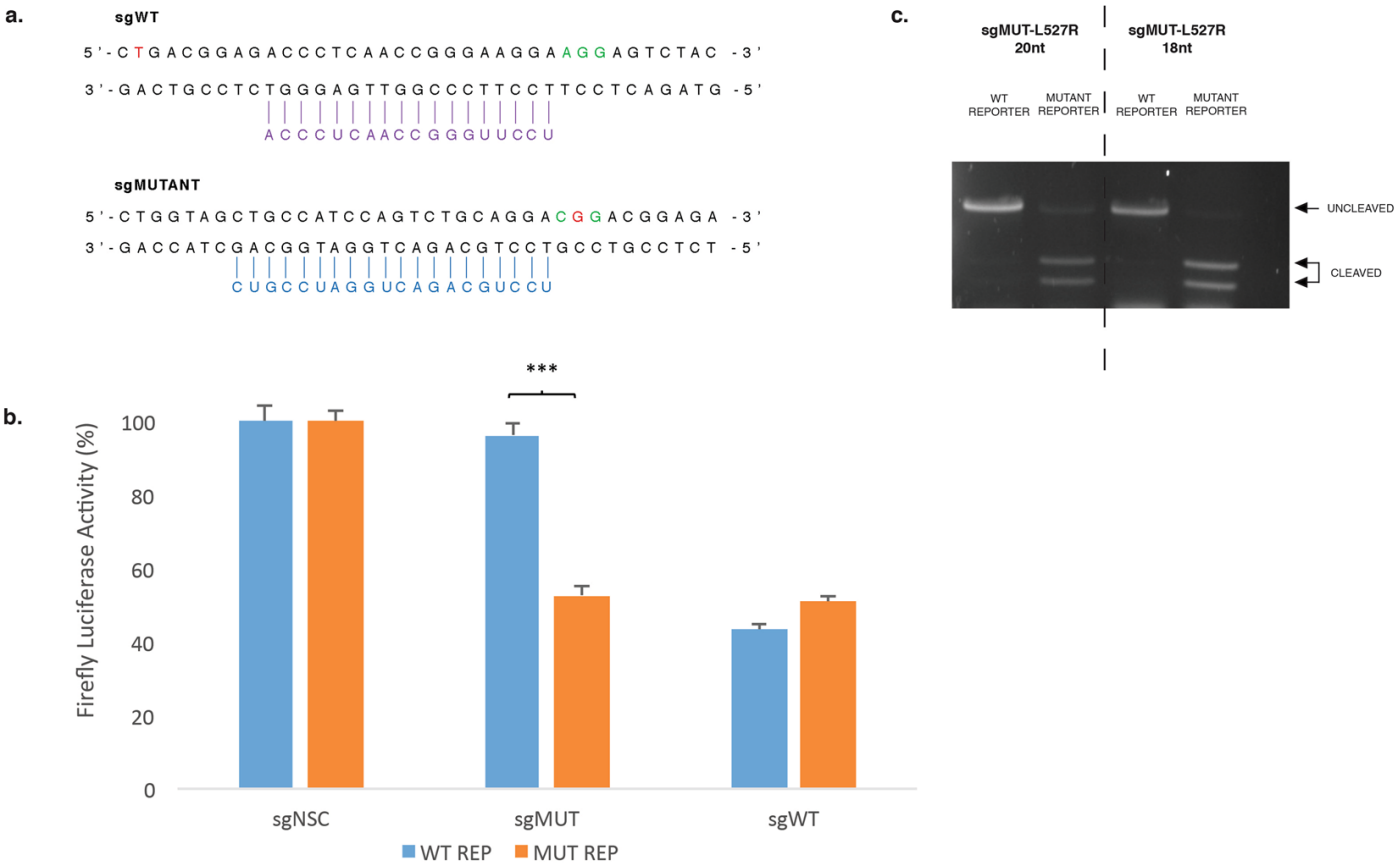
Allele-specific cleavage of L527R *TGFBI* mutation utilising a PAM-specific approach. (**a**) The L527R mutation (c.1580 T > G) is indicated in red and PAM utilised is shown in green. A 20 nt sgRNA targeted to a naturally occurring PAM was designed as a positive control (sgWT, purple –top of figure). A 20 nt sgRNA utilising the novel PAM, containing the L527R mutation, was designed (sgMUTANT, blue – bottom of figure). (**b**) Both sgWT and sgMUTANT were targeted to a luciferase reporter plasmid containing either a wild-type or mutant *TGFBI* sequence to determine potency and allele specificity. (**c**) An *in vitro* digestion with Cas9 protein complexed with a sgRNA utilising the novel L527R PAM was carried out to confirm the specificity observed. Mutant guides of both 20 and 18 nucleotides were tested. Uncropped gel images are available in Supplementary Figure 1.

A PAM-specific approach has previously been shown to be an ideal way to achieve allele-specific editing^14^. To validate this approach a lattice corneal dystrophy-associated *TGFBI* mutation (L527R), was assessed^33^. The L527R mutation (c.1580 T > G) generates a novel PAM with *S. pyogenes*, (CTG > CGG) (Fig. 4a, top). A 20 nt sgRNA utilising the novel PAM was designed and an additional 20 nt sgRNA targeted to a naturally occurring PAM was designed as a positive control (Fig. 4a, bottom). Specificity was first assessed using a previously described *in vitro* dual-luciferase assay^7,9,34^ in which the two sgRNAs were co-expressed with either *S. pyogenes* Cas9, *S.aureus* Cas9 or *As*Cpf1 and a luciferase reporter containing a 50 bp region of either wild -type or mutant *TGFBI* sequence, which has been cloned into the multiple-cloning-site within the 3′UTR of *Luc2*. Cleavage of the *TGFBI* sequence within the reporter construct prevents transcription and processing of luciferase mRNA and results in an proportionate reduction of luciferase expression and therefore luciferase activity was measured as an indicator of sgRNA activity. The sgRNA utilising the novel PAM was shown to be highly specific, directing cutting of only the mutant *TGFBI* sequence, while both reporters were cleaved by the common sgRNA (Fig. 4b). In addition, an *in vitro* digestion using mutant 18 and 20 nt sgRNAs with a reporter containing either wild-type or mutant *TGFBI* sequence was carried out which confirmed the specificity observed in the dual-luciferase assay (Fig. 4c). Co-transfection with the mutant 18 and 20 nt sgRNAs only resulted in cleavage of the mutant reporter, the wild-type reporter template remained intact. Truncation of the guide did not appear to improve specificity.

### Investigation of Cas9 orthologues *S.aureus* and *AsCpf1*

**Figure 5:**
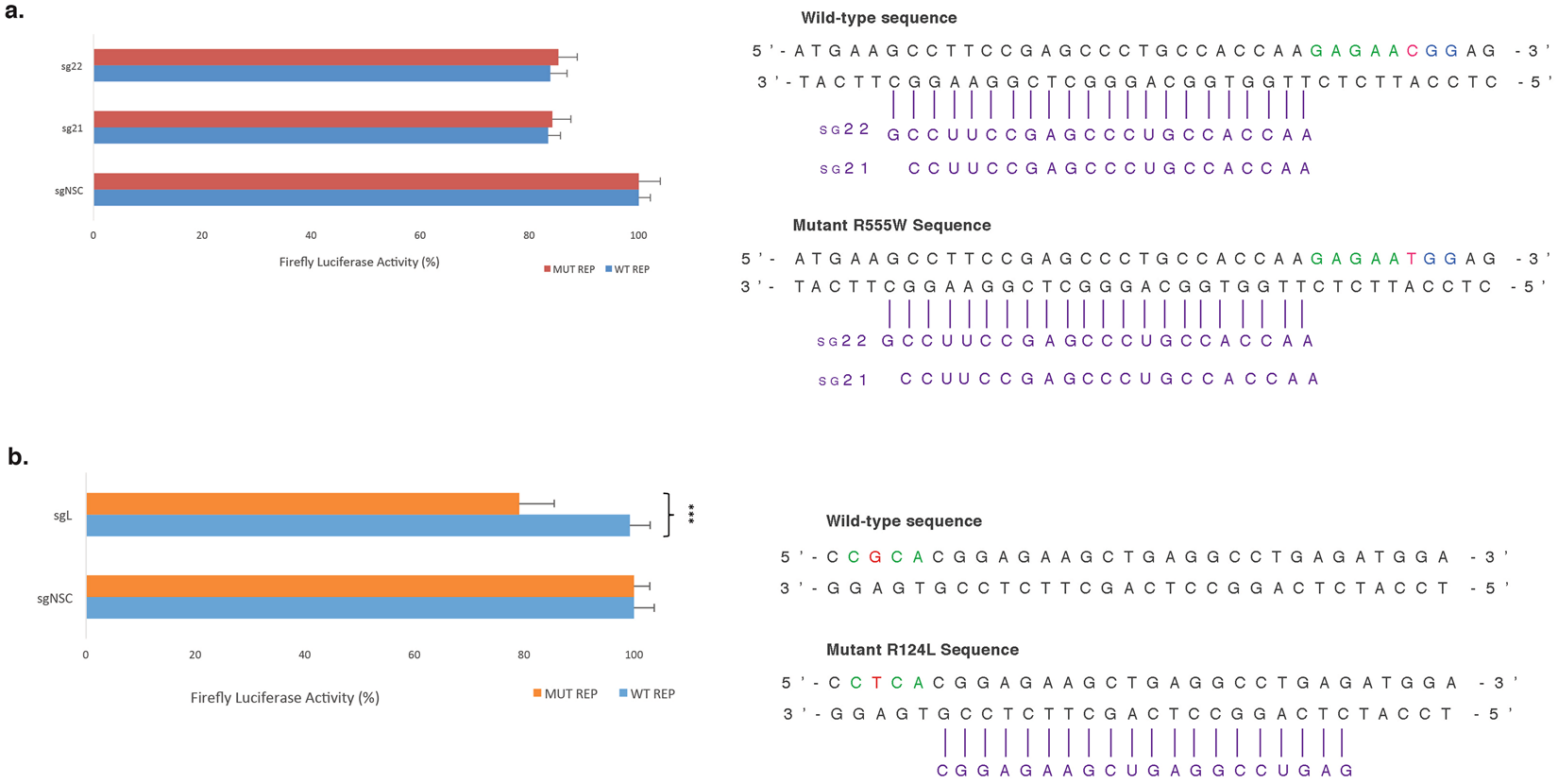
Evaluation of Cas9 orthologues in a PAM-specific system targeted to prevalent *TGFBI* mutations Guide RNA tested shown in purple, PAM utilised shown in green and mutation shown in red. (**a**) 22 and 21 nucleotide guides were designed to target the novel *S.aureus* Cas9 PAM generated by R555W. Both guide lengths were targeted to a luciferase reporter plasmid containing either a wild-type or mutant *TGFBI* sequence to determine potency and allele specificity. (**b**) A guide utilising the novel mutant *As*Cpf1 PAM generated by R124L was targeted to a luciferase reporter plasmid containing either a wild-type or mutant *TGFBI* sequence to determine potency and allele specificity.

As none of the prevalent TGFBI mutations generated a novel PAM with *S. pyogenes* Cas9, alternative Cas9 orthologues were investigated. Although *S.aureus* Cas9 prefers a 5′-NNGRRT-3′ PAM, generated by the *TGFBI* R555W mutation (5′-GAGAAT-3′) (Fig. 3b). It has also been shown to recognise a 5′-NNGRRV-3′ PAM with comparable efficiencies^35^, and this is present in the wild-type *TGFBI* sequence. Since *S.aureus* Cas9 prefers a guide length of either 21 nucleotides or 22 nucleotides^13^. both 21 nt and 22 nt guides utilising the novel *S.aureus* PAM were designed and targeted to both wild-type and mutant R555W *TGFBI* sequences. No significant knockdown was observed with either guide length and the mutant R555W guide was unable to distinguish between wild-type and mutant *TGFBI* sequence (Fig. 5a).

A mutant *As*Cpf1 was generated that has the capability of recognising a 5′-VYVC-3′ PAM, as generated by the *TGFBI* R124L mutation (5′-CTCA-3′) (Fig. 3b). A 20 nt guide was designed utilising the novel mutant *As*Cpf1 PAM and targeted to both the wild-type and mutant R124L TGFBI sequences. Although the mutant guide can distinguish between wild-type and mutant *TGFBI* sequence the knockdown efficiency is very low with a maximal knockdown of 20% (Fig. 5b).

### Investigation of a guide-specific approach using *S. pyogenes* Cas9

**Figure 6:**
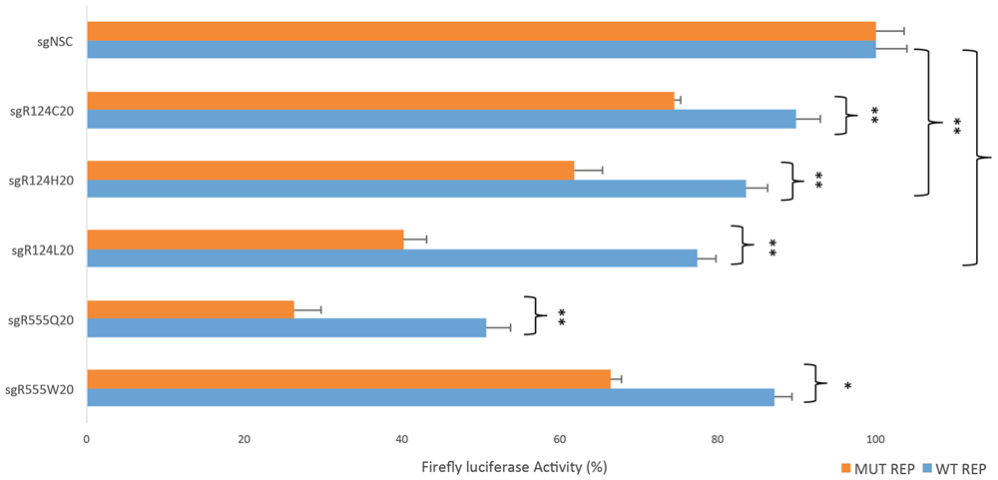
Investigation of a guide-specific approach to treat prevalent *TGFBI* mutations Using a guide-specific approach, 20 nucleotide guides for the 5 most prevalent TGFBI mutations (as shown in Fig. 3a) were targeted to wild-type and respective mutant sequence in a dual luciferase assay. The 5 guides cut with varying degrees of specificities and efficiencies. There was a significant difference between the wild-type and mutant sequence in all cases.

As none of the most prevalent *TGFBI* mutations generated a novel PAM with *S. pyogenes* Cas9 and adequate specificity or efficiency could not be achieved with Cas9 orthologues from other bacterial species, a guide-specific approach was explored; whereby the mutant guide differs from the wild-type sequence only by a single base pair. A dual-luciferase assay was employed to assess the specificity of a 20 nt guide for the 5 most prevalent TGFBI mutations; R124C, R124H, R124L, R555Q and R555W. Each guide was targeted to the wild-type and respective mutant sequence and the firefly luciferase activity was measured as an indicator of specificity (Fig. 6).

Cas9 directed by R124C sgRNA was able distinguish between wild-type and mutant sequence, although it cut with a low efficiency of 26%. R124H cut with an improved specificity and efficiency, although wild-type sequence was significantly cleaved (17%). R124L offered the most promising specificity profile, 60% cleavage of mutant sequence was observed in comparison to 23% of the wild-type sequence, however the wild-type sequence was still significantly cleaved when compared to the non-specific control. Although the R555Q guide directed efficient cleavage of the mutant reporter, the wild-type sequence was also substantially cut by 50%. Finally R555W preferentially cleaved mutant sequence, however the wild-type sequence was still cleaved by 10%.

### Investigation of the effect of guide length on the specificity of *S. pyogenes* Cas9

**Figure 7:**
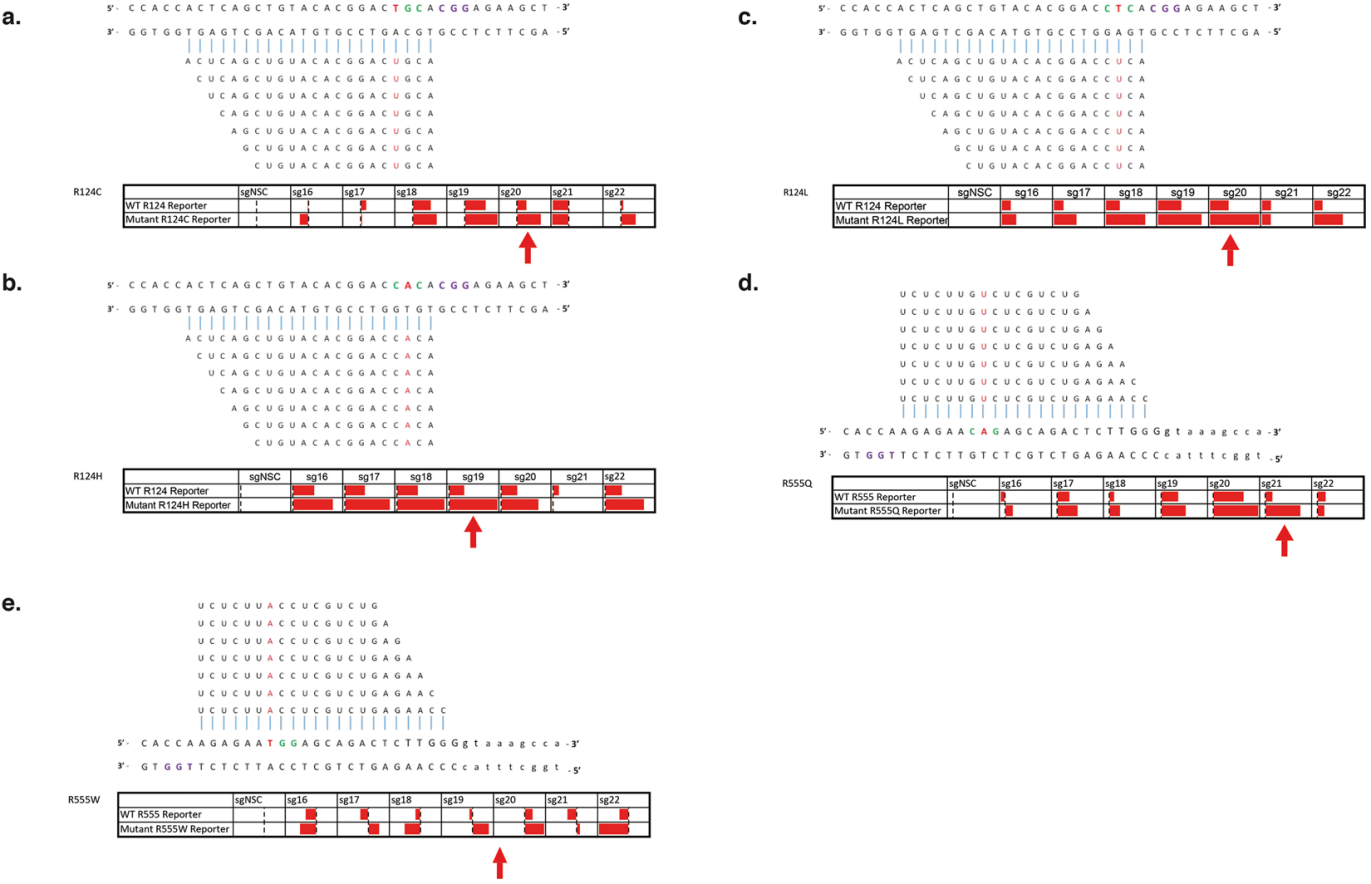
Guide-length screen to determine the effect on specificity of a guide-specific system Heatmaps showing varying degrees of knockdown observed via a dual luciferase assay when guides ranging in different lengths are targeted to the wild-type and respective mutant sequence. Specificity bars show knockdown when normalised to the non-specific control, with 100% being maximal knockdown observed. Maximal allele-specificity observed for each mutation indicated with a red arrow.

Reports have indicated that truncating the length of the matching sequence within the guide to 18 nucleotides can reduce off-target cutting, while maintaining on-target efficiencies^12^. As none of the 20 nt guides provided adequate specificity an assessment of the effect of guide-length upon specificity using a dual-luciferase assay was conducted for the 5 most prevalent TGFBI mutations. Reports have shown that guide lengths <16 nt abolish cleavage activity^36,37^. For each mutation a range of guide lengths from 16–22 nucleotides were tested, each guide was targeted to the wild-type and respective mutant sequence and the firefly luciferase activity was measured as an indicator of specificity (Fig. 7).

For all mutations investigated the truncated guides did not provide a marked improvement of specificity, for most cases maximal discrimination occurred with guides 20 or 19 nucleotides in length. For R124C, a 20 nt guide seemed to confer allele-specificity, however no other guide length offered any adequate discrimination (Fig. 7a). In the case of the R555Q mutation guides in the 18–20 nt range did not offer sufficient discrimination, although, interestingly, the 21 nt guide provided convincing allele-specificity (Fig. 7d). R555W did not offer any considerable allele-specificity for any length tested (Fig. 7e). R124H and R124L displayed clear allele-specific cleavage, especially in the 18–20 nt sgRNA range, with minimal cutting of the wild-type sequence (Fig. 7b,c). Interestingly for the R124 mutations guide lengths of 21 nt seemed to impair cleavage activity in all cases.

### Addition of 5′-GG to the 20 nt guide sequence

**Figure 8:**
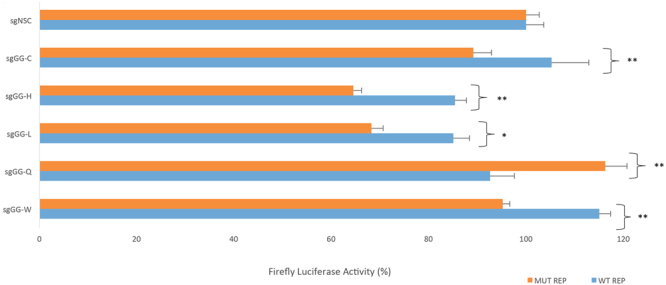
Effect of the addition of 5′-GG to the 20 nt guide sequence on specificity 5′-GG was prefixed to the 20 nucleotide guides for the 5 most prevalent TGFBI mutations (as shown in Fig. 3a) were targeted to wild-type and respective mutant sequence in a dual luciferase assay. The addition of 5′-GG did not improve specificity, in some cases it caused a reduction in on-target activity.

Standard design of sgRNA guides includes the addition of a guanine to the 5′ end of the guide sequence (5′-GX_20_-3′) to help facilitate efficient transcription^4^. An alternative guide design of 5′-GGX_20_-3′ has been reported to minimise off-target activity in certain cases, offering an improved specificity of Cas9^38^. This parameter was tested using the 5 most prevalent TGFBI mutations (Fig. 8). The additional guanine at the 5′ end of the guide sequence did not provide an improved specificity in any case. In some instances a reduction in on-target activity was observed, confirming that specificity is guide dependent.

### *In vitro* digestion to confirm specificity of *S. pyogenes* Cas9

**Figure 9:**
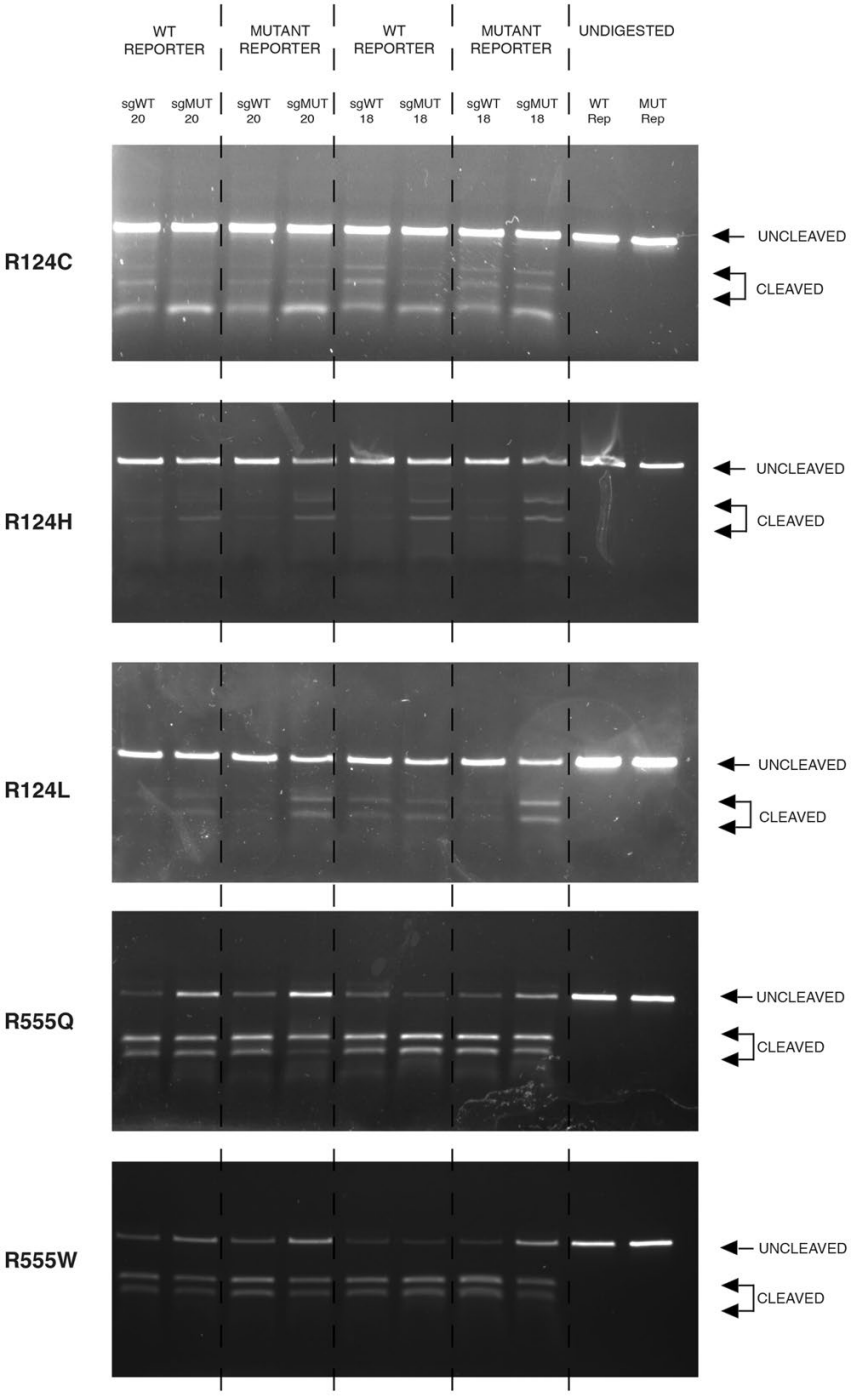
Confirmation of the specificity achieved using a guide-specific system targeted to prevalent *TGFBI* mutations *In vitro* digestion of either wild-type or respective mutant *TGFBI* sequence via Cas9 protein complexed with an sgRNA. Guides lengths of 20 and 18 nucleotides were assessed. Uncropped gel images are available in Supplementary Figure 2.

*In vitro* digestion of either wild-type or mutant *TGFBI* sequence with Cas9 protein complexed with sgRNA was carried out to further assess the specificity profile of *S. pyogenes* Cas9 (Fig. 9). Guide lengths of 18 and 20 nucleotides were tested to evaluate the impact of truncating the guide sequence. For R124C the mutant 20 nt guide appeared to cut the mutant sequence more than the wild-type sequence. However, when truncated to 18 nt the mutant guide appeared to loose ability to distinguish between wild-type and mutant sequence, reflecting results from the dual luciferase assay. For R124H and R124L both mutant 20 nt and 18 nt guides appeared to clearly cut the mutant sequence preferentially over the wild-type sequence, again reflecting the dual-luciferase results. Interestingly, in both cases the wild-type guide appeared to result in more cleavage of the mutant sequence in comparison to the mutant sequence, although as the wild-type guide would not be implicated in a clinical setting it can be ignored. For R555Q and R555W the 20 nt or 18 nt guides did not confer allele-specificity under any conditions, cutting both wild-type and mutant sequence equally, demonstrating mismatches in the distal region of the guide are less critical in determining specificity of Cas9.

## Discussion

Dominant negative disorders that are the result of an accumulation of mutant protein can be targeted by allele-specific CRISPR mediated gene disruption via NHEJ. We have shown *in vivo* that gene disruption via NHEJ offers a viable approach to achieve gene silencing. Sustained knockdown of luciferase was observed in the corneal epithelium of reporter mice over several weeks in 3 out of 4 mice, following a single intrastromal injection of CRISPR/Cas9 components (Fig. 2c). Since the corneal epithelium is completely turned over every 1–2 weeks^39^, our data suggests permanent editing took place within the corneal stem cell compartment following *in vivo* delivery of CRISPR/Cas9. By extension, CRISPR/Cas9 gene editing using an sgRNA specific to pathologic mutant alleles delivered by intrastromal injection has great potential for editing resident corneal stem cells as a permanent cure for dominant-negative corneal disorders. However, in order to translate this strategy to the clinic as a therapy the issue of specificity must be addressed.

The prevalent *TGFBI* mutations offer an interesting real-life scenario in which to test different approaches to allele-specific CRISPR/Cas9 gene therapy as the different causative mutations with different phenotypes associated with the same codon create different specificity profiles.

Published reports illustrate that the region immediately adjacent to the PAM is critical to specificity^25–27^. The documented importance of this region has led to it being coined as the ‘seed’ region. The Cas9:sgRNA complex will initially identify the correct PAM, and only once the PAM has been identified will the Cas9:sgRNA complex then test the complementarity between the guide and target DNA. The PAM proximal region, or seed region, is critical in this step and mismatches in this region will prevent the ternary complex forming and therefore cleavage will not occur^40^. The exact length of the seed region is unclear, with reports ranging from 5–12 nucleotides^29–31^.

The *TGFBI* mutations investigated here gave the opportunity to investigate the extent of this seed region further. The mutations in codon 124 lie at guide positions 3 or 4, so are within the seed region of whichever definition, whereas, codon 555 mutations lie in guide positions 7 or 8, so can be considered either inside or outside the seed region. Accordingly, it was demonstrated that allele-specificity was achieved by guides targeting R124H and R124L mutations both found at position 3 of the guide. However, neither R124C, R555Q or R555W mutations found at positions 4, 7 and 8 respectively, were capable of adequate allele-specific cleavage. This confirms that the sequence immediately adjacent to the PAM is most critical in determining specificity of the guide, and mismatches are not well tolerated here. In contrast mismatches in positions 4, 7 or 8 of the guide are better tolerated and do not have as strong an influence on the fidelity of the guide^41^.

In addition, it has been demonstrated that U-rich seeds are linked with a low knockdown efficiency, due to RNA polymerase III being terminated by U-rich sequences. Interestingly, the R555W mutation in which minimal knockdown was observed has a very U-rich seed with 4 U’s within the first 6 bp: 3′-UCUCUU-5′^30^. Jiang *et al*. reported that mutations in positions ranging from position 1 in the guide to position 6 of the guide sequence abolish cleavage activity, except mutations at position 3^41^. These *TGFBI* results directly contradict this, as the R124H and R124L mutations exhibit clear allele-specific cleavage and both mutations are present at position 3 of the guide. Therefore, it is evident that restrictions most likely vary from one guide sequence to another and in each case should be individually assessed.

That single mismatches in guide sequences can be tolerated regardless of their position in the guide has been confirmed in other reports^11,42,38^. Contrary to initial reports using other genes, manipulation of the guide sequence, in the form of truncation or addition of extra guanine bases, did not provide improved specificity in any case. This is consistent with follow up reports that demonstrate truncated guides or additional guanines do not offer improved specificity in most cases^42,43^. An intriguing observation was that for all R124 mutations the guide length of 21 nt seemed to impair cleavage activity, it is unclear why this happens but we hypothesise it may alter structure of stability of the sgRNA.

To confirm whether the results observed *in vitro* could be directly translated to a real-life scenario, it would be compelling to test these guides *ex vivo* in patient derived primary cells or *in vivo* in a mutation-specific animal model. This would demonstrate the effectiveness of a combined *in vitro* dual-luciferase and cleavage assay as a preliminary screening stage to ensure guides with adequate specificity are utilised downstream in a clinical setting.

The use of CRISPR therefore has clear limitations in targeting specific disease-causing mutations. In circumstances when one is not tied to targeting a specific disease-causing mutation, the criteria for selecting an appropriate sgRNA can be outlined as; avoid selecting guides that have predicted off-targets directly followed by a PAM, high global sequence similarity, mismatches only in the PAM distal region and those that do not have maximal consecutive mismatches^10^. However, when designing an sgRNA to targeting a particular disease-causing mutation there is no flexibility (other than guide length) to meet these criteria. Consequently, a guide-specific treatment strategy is not suitable for targeting the mutant alleles which cause *TGFBI* corneal dystrophies, as an almost perfect off-target site exists in the form of the wild-type allele.

Although here, and in previous reports, a SNP-derived PAM approach has been shown to provide highly specific cleavage^15^, this can only be applied to PAM-generating mutations. In the case of *TGFBI* corneal dystrophies, of the 60 causative mutations less than a third generate a novel *S. pyogenes* PAM. Therefore, even if the problems associated with Cas9/sgRNA delivery at present were overcome, the majority of patients with TGFBI would not have mutations that could be directly targeted.

Cas9 *S. pyogenes* orthologues are not as well characterised, therefore their off-target profiles are not as well understood as the that of *S. pyogenes*. In addition to this, they have much more intricate PAMs that occur much less frequently in the genome, reducing the fraction of *TGFBI* mutations that will result in a novel PAM. Furthermore, our results highlight another concern; even though the mutant SNP generated a novel PAM a non-canonical PAM existed in the wild-type sequence (Fig. 5b), meaning allele-specific cleavage could not be achieved. If non-canonical PAMs are considered within the analysis the number of targetable mutations would be even further reduced.

It is clear that individual guides perform with different cleavage efficiencies and specificity profiles. It is unrealistic to suggest a 60 allele-specific guide system as an effective treatment for *TGFBI* corneal dystrophies. A need for a highly-specific catch-all approach is apparent.

## Materials and Methods

### Oligonucleotides

All oligonucleotides used in this study were purchased from Integrated DNA Technologies. Sequences are listed in Supplementary Table 2.

### Constructs

The *S. pyogenes* Cas9 vector plasmid used was pSpCas9(BB)-2A-Puro (PX459) V2.0, a gift from Feng Zhang (Addgene plasmid # 62988).The *S.aureus* Cas9 vector plasmid used was pX601-AAV-CMV::NLS-SaCas9-NLS-3xHA-bGHpA;U6::BsaI-sgRNA, a gift from Feng Zhang (Addgene plasmid # 61591). The mutant *AsCpf1* used was kindly provided from Professor Feng Zhang, Broad Institute MIT. Wild-type *TGFBI* or mutant *TGFBI* guides were cloned into the various plasmids by standard molecular biology techniques. A detailed protocol is outlined by Ran *et al*.^4^. In brief, *S. pyogenes* Cas9 and mutant *As*Cpf1 were digested with *BbsI* (NEB Cat # R0539S) while *S.aureus* Cas9 was digested with *BsaI* (NEB Cat # R0535S). Guide sequences (shown in Supplementary Table 2) were annealed and cloned into the corresponding digested plasmid.

A firefly luciferase reporter plasmid was used to assess knockdown. The vector plasmid used was psiTEST-LUC-Target (York Bioscience Ltd, York, UK). 50 nucleotides of wild type *TGFBI* or mutant *TGFBI* sequence was cloned into the MCS by standard molecular biology techniques.

An expression construct for Renilla luciferase (pRL-CMV, Promega, Southampton, UK) was used for the dual-luciferase assay to normalize transfection efficiency. In brief, psiTEST-LUC-Target was digested with NheI and KpnI (NEB Cat # R0131S and # R0142S). Human wild-type or mutant *TGFBI* sequences (shown in Supplementary Table 2) were annealed and cloned into the digested plasmid.

### Off-target analysis

Off-target and on-target scores were calculated using the ‘Optimised CRISPR Design Tool’, available online by the Zhang lab, MIT 2013 and ‘Benchling’s CRISPR Tool’ available online by Benchling.

### Dual-Luciferase Assay

A dual luciferase assay was used to determine the potency and allele specificity of the different guides previously described. HEK AD293 cells (Life Technologies) were co-transfected using Lipofectamine 2000 (Life Technologies) with a CRISPR plasmid, a firefly luciferase reporter plasmid and *Renilla* Luciferase expression plasmid. Cells were incubated for 72 hours, before being lysed and the activities of both *Firefly* luciferase and *Renilla* luciferase quantified.

### Intrastromal Injection

Animals were used for the following experiments in accordance with the UK Animal Welfare Act; the experiments were approved by the Home Office (Scotland) and the DHSSPS (Northern Ireland). Prior to intrastromal injection of CRISPR components, mice were anaesthetised by intraperitoneal injection with a mix of Hypnorm (25 mg/kg; VetaPharma Ltd, Leeds, UK) and Hypnovel (25 mg/kg; Roche, Hertfordshire, UK). In addition, topical anaesthetic (0.5%w/v Tetracaine Hydrochloride; Bausch & Lomb, Aubenas, France) was applied to the eye. Following injection, mice were allowed to recover in a heated cabinet and monitored for adverse effects until the anesthesia had worn off fully. Cas9/sgRNA constructs were delivered to the mouse cornea by intrastromal injection, as previously described (Courtney *et al*.^8^). Both a guide targeted to *Luc2* (sgLuc2 - right eye) and a non-specific control guide (sgNSC - left eye) were injected intrastromally in a total volume of 4 µl of PBS at a concentration of 500ng/µl.

### Live animal imaging

All mice used for live imaging were aged between 12 and 25 weeks old. For imaging, mice were anaesthetised using 1.5–2% isoflurane (Abbott Laboratories Ltd., Berkshire, UK) in ~1.5 l/min flow of oxygen. A mix of luciferin substrate (30 mg/ml D-luciferin potassium salt; Gold Biotechnology, St. Louis, USA) mixed 1:1 w/v with Viscotears gel (Novartis, Camberley, UK) was dropped onto the eye of heterozygous Krt12 +/luc2 transgenic mice immediately prior to imaging. A Xenogen IVIS Lumina (Perkin Elmer, Cambridge, UK) was used to quantify luminescence. Live images of mice (n = 4) were taken every 24 hours for 7 days, then once every week thereafter for six weeks (42 days) in total. Quantification of luciferase inhibition was determined by calculating the right/left ratio, with values normalised to those at day 0 (as 100%).

### *In vitro* digestion of circular plasmid and DNA template with purified *S. pyogenes* Cas9

A double-stranded DNA template was prepared by amplifying a region of the luciferase reporter plasmid containing the desired sequence using the following primers: 5′-ACCCCAACATCTTCGACGCGGGC-3′ and 3′-TGCTGTCCTGCCCCACCCCA-5′. A cleavage reaction was set up by incubating 30 nM *S. pyogenes* Cas9 nuclease (NEB UK) with 30 nM synthetic sgRNA (Synthego) for 10 minutes at 25 °C. The Cas9:sgRNA complex was then incubated with 3 nM of DNA template at 37 °C for 1 hour. Fragment analysis was then carried out on a 1% agarose gel.

### Statistical analysis

All error bars represent the S.E.M. unless stated otherwise. Significance was calculated using a Mann-Whitney test. Statistical significance wasset at p < 0.05. Variance was calculated among groups and deemed to be similar.

### Data availability

No datasets were generated or analysed during the current study.

## Electronic supplementary material

Supplementary Information
