# Supplementary material for: Towards personalised allele-specific CRISPR gene editing to treat autosomal dominant disorders
Source: Sci Rep. 2017 Nov 23;7:16174. doi: 10.1038/s41598-017-16279-4 (PMC5701044; doi:10.1038/s41598-017-16279-4)

Towards personalised allele-specific CRISPR gene editing to treat autosomal dominant disorders

Kathleen A. Christie,[1] David G. Courtney,[1] Larry A. DeDionisio,[2] Connie Chao Shern, [1] Shyamsree De Majumdar,[1] Laura C. Mairs[1], M. Andrew Nesbit,[1] C.B.Tara Moore[1]*

Supplementary Table 1: Mutational analysis performed on the TGFBI corneal dystrophy mutations to determine which i) generated a novel *S.pyogenes* PAM or ii) had a near-by *S.pyogenes* PAM

	Lattice Type			Wild-type *TGFBI* sequence	Mutant *TGFBI* sequence	Novel *S.pyogenes* PAM	Novel *S.aureus* PAM	Near-by *S.pyogenes* PAM	Near-by *S.aureus* PAM
Missense	R496W	C>T	CGG>TGG	GTACGGGACCCTGTTCACGATGGACCGGGTGCTGACCCCCCCAATGGGGAC	GTACGGGACCCTGTTCACGATGGACTGGGTGCTGACCCCCCCAATGGGGAC			X (position 1)	X (position 13)
	R514P	G>C	CGC>CCC	ATGGATGTCCTGAAGGGAGACAATCGCTTTAGgtaattagttccatccccg	ATGGATGTCCTGAAGGGAGACAATCCCTTTAGgtaattagttccatccccg	X		X (position 5)	
	F515L	T>A	TTT>TTA	ATGTCCTGAAGGGAGACAATCGCTTTAGgtaattagttccatccccgggtg	ATGTCCTGAAGGGAGACAATCGCTTAAGgtaattagttccatccccgggtg			X (position 1)	X (position 19)
	T538P	A>C	ACA>CCA	GACCCTCAACCGGGAAGGAGTCTACACAGTCTTTGCTCCCACAAATGAAGC	GACCCTCAACCGGGAAGGAGTCTACCCAGTCTTTGCTCCCACAAATGAAGC	X		X (position 14)	X (position 19)
	V539D	T>A	GTC>GAC	CCTCAACCGGGAAGGAGTCTACACAGTCTTTGCTCCCACAAATGAAGCCTT	CCTCAACCGGGAAGGAGTCTACACAGACTTTGCTCCCACAAATGAAGCCTT			X (position 18)	
	F540S	T>C	TTT>TCT	CAACCGGGAAGGAGTCTACACAGTCTTTGCTCCCACAAATGAAGCCTTCCGA	CAACCGGGAAGGAGTCTACACAGTCTCTGCTCCCACAAATGAAGCCTTCCGA				
	P542R	C>G	CCC>CGC	GAAGGAGTCTACACAGTCTTTGCTCCCACAAATGAAGCCTTCCGAGCCC	GAAGGAGTCTACACAGTCTTTGCTCGCACAAATGAAGCCTTCCGAGCCC				
	A546D	C>A	GCC>GAC	CACAGTCTTTGCTCCCACAAATGAAGCCTTCCGAGCCCTGCCACCAAGAGA	CACAGTCTTTGCTCCCACAAATGAAGACTTCCGAGCCCTGCCACCAAGAGA			X (position 10)	X (position 20)
	F547S	T>C	TTC>TCC	AGTCTTTGCTCCCACAAATGAAGCCTTCCGAGCCCTGCCACCAAGAGAACG	AGTCTTTGCTCCCACAAATGAAGCCTCCCGAGCCCTGCCACCAAGAGAACG	X		X (position 1)	
	R548P	G>C	CGA>CCA	TCCCACAAATGAAGCCTTCCGAGCCCTGCCACCAAGAGAACGGAG	TCCCACAAATGAAGCCTTCCCAGCCCTGCCACCAAGAGAACGGAG	X		X (position 4)	
	P551Q	C>A	CCA>CAA	ACAAATGAAGCCTTCCGAGCCCTGCCACCAAGAGAACGGAGCAGACTCTTG	ACAAATGAAGCCTTCCGAGCCCTGCAACCAAGAGAACGGAGCAGACTCTTG			X (position 3)	
	L558R	T>G	CTC>CGC	GCCACCAAGAGAACGGAGCAGACTCTTGGgtaaagaccaacttaagtacacgtc	GCCACCAAGAGAACGGAGCAGACGCTTGGgtaaagaccaacttaagtacacgtc			X (position 3)	X (position 2)
	L558P	T>C	CTC>CCC	CTGCCACCAAGAGAACGGAGCAGACTCTTGGgtaaagaccaacttaagtac	CTGCCACCAAGAGAACGGAGCAGACCCTTGGgtaaagaccaacttaagtac	X		X (position 3)	X (position 2)
	L565P	T>C	CTT>CCT	GAGATGCCAAGGAACTTGCCAACATCCTGAAATACCACATT	GAGATGCCAAGGAACCTGCCAACATCCTGAAATACCACATT	X		X (position 7)	
	G594V	G>T	GGT>GTT	CTGGTGCGGCTAAAGTCTCTCCAAGGTGACAAGCTGGAAGTCAGCTTGgta	CTGGTGCGGCTAAAGTCTCTCCAAGTTGACAAGCTGGAAGTCAGCTTGgta			X (position 3)	
	V613G	G>T	GTT>GGT	TGTGGTGAGTGTCAACAAGGAGCCTGTTGCCGAGCCTGACATCATGGCCAC	TGTGGTGAGTGTCAACAAGGAGCCTGGTGCCGAGCCTGACATCATGGCCAC	X		X (position 2)	
	A620D	C>A	GCC>GAC	CCTGTTGCCGAGCCTGACATCATGGCCACAAATGGCGTGGTCCATGTCATC	CCTGTTGCCGAGCCTGACATCATGGACACAAATGGCGTGGTCCATGTCATC			X (position 7)	
	V624M	G>A	GTG>ATG	CTGACATCATGGCCACAAATGGCGTGGTCCATGTCATCACCAATGTTCTG	CTGACATCATGGCCACAAATGGCATGGTCCATGTCATCACCAATGTTCTG			X (position 1)	
	V625D	G>A	GTC>ATC	CTGACATCATGGCCACAAATGGCGTGGTCCATGTCATCACCAATGTTCTG	CTGACATCATGGCCACAAATGGCGTGATCCATGTCATCACCAATGTTCTG			X (position 12)	
Small Deletions	His572del		c.1714_1716delCAC	GGAACTTGCCAACATCCTGAAATACCACATTGGTGATGAAATCCTGGTTAG	GGAACTTGCCAACATCCTGAAATAC_ATTGGTGATGAAATCCTGGTTAG			X (position 3)	X (position 7)
	Val613del		c.1838_1849del12	TGTGGTGAGTGTCAACAAGGAGCCTGTTGCCGAGCCTGACATCATGGCCACA	TGTGGTGAGTGTCAACAAGGAGCCTG_ACATCATGGCCACAAAATGGCGTGGTC			X (position 2)	
	Val624_Val625del		c.1870_1875delGTGGTC	CTGACATCATGGCCACAAATGGCGTGGTCCATGTCATCACCAATGTTCTGAGCCTC	CTGACATCATGGCCACAAATGGC_CATGTCATCACCAATGTTCTGCAGCCTC			X (position 9)	
	**Lattice Intermediate type I/IIIA**								
Missense	L518R	T>C	CTG>CCG	ttctgtgtgtgtatctacagCATGCTGGTAGCTGCCATCCAGTCTGCAGGA	ttctgtgtgtgtatctacagCATGCCGGTAGCTGCCATCCAGTCTGCAGGA	X			
	L518P	T>G	CTG>CGG	ttctgtgtgtgtatctacagCATGCTGGTAGCTGCCATCCAGTCTGCAGGA	ttctgtgtgtgtatctacagCATGCGGGTAGCTGCCATCCAGTCTGCAGGA	X	X		
	T538R	C>G	ACA>AGA	GACCCTCAACCGGGAAGGAGTCTACACAGTCTTTGCTCCCACAAATGAAGCTT	GACCCTCAACCGGGAAGGAGTCTACAGAGTCTTTGCTCCCACAAATGAAGCC		X	X (position 15)	X (position 20)
	N544S	A>G	AAT>AGT	GTCTACACAGTCTTTGCTCCCACAAATGAAGCCTTCCGAGCCCTGCCACCA	GTCTACACAGTCTTTGCTCCCACAAGTGAAGCCTTCCGAGCCCTGCCACCA			X (position 4)	
	N622H	A>C	AAT>CAT	GCCGAGCCTGACATCATGGCCACAAATGGCGTGGTCCATGTCATCACCAAT	GCCGAGCCTGACATCATGGCCACACATGGCGTGGTCCATGTCATCACCAAT			X (position 2)	
	G623D	G>A	GGC>GAC	GAGCCTGACATCATGGCCACAAATGGCGTGGTCCATGTCATCACCAATGTT	GAGCCTGACATCATGGCCACAAATGACGTGGTCCATGTCATCACCAATGTT			X (position 3)	
	H626R	A>G	CAT>CGT	CATCATGGCCACAAATGGCGTGGTCCATGTCATCACCAATGTTCTG	CATCATGGCCACAAATGGCGTGGTCCGTGTCATCACCAATGTTCTG			X (position 16)	
	H626P	A>C	CAT>CCT	CATCATGGCCACAAATGGCGTGGTCCATGTCATCACCAATGTTCTG	CATCATGGCCACAAATGGCGTGGTCCCTGTCATCACCAATGTTCTGCAGCCTC	X		X (position 16)	
Small Deletion	c.1618_1620delTTT		Phe540del	CAACCGGGAAGGAGTCTACACAGTCTTTGCTCCCACAAATGAAGCCTTCCGA	CAACCGGGAAGGAGTCTACACAGTC_GCTCCCACAAATGAAGCCTTCCGA			X (position 20)	
Small Insertion	c.1886_1894dupCCAATGTTC		Thr629_Asn630insAsnValPro	AATGGCGTGGTCCATGTCATCACCAATGTTCTGCAGCCTCCAGgtaagtgtcgcatcc	CATGTCATCACCAATGTTCCCAATGTTCTGCAGCCTCCAGgtaagtgtcgcatcc	X			
	**Lattice Type I**								
Missense	R124C	C>T	CGC>TGC	CACCACCACTCAGCTGTACACGGACCGCACGGAGAAGCTGAGGCCTGAGAT	CACCACCACTCAGCTGTACACGGACTGCACGGAGAAGCTGAGGCCTGAGAT			X (position 4)	X (position 13)
	V505D	T>A	GTC>GAC	GTGCTGACCCCCCCAATGGGGACTGTCATGGATGTCCTGAAGGGAGACAAT	GTGCTGACCCCCCCAATGGGGACTGACATGGATGTCCTGAAGGGAGACAAT			X (position 3)	X (position 2)
	I522N	T>A	ATC>AAC	atctacagCATGCTGGTAGCTGCCATCCAGTCTGCAGGACTGACGGAGACC	atctacagCATGCTGGTAGCTGCCAACCAGTCTGCAGGACTGACGGAGACC			X (position 1)	
	L569R	T>G	CTG>CGG	GATGCCAAGGAACTTGCCAACATCCTGAAATACCACATTGGTGATGAAATC	GATGCCAAGGAACTTGCCAACATCCGGAAATACCACATTGGTGATGAAATC	X		X (position 7)	
	L569Q	T>G	CTG>CGG	ATGCCAAGGAACTTGCCAACATCCTGAAATACCACATTGGTGATGAAATCC	ATGCCAAGGAACTTGCCAACATCCGGAAATACCACATTGGTGATGAAATCC	X		X (position 7)	
	H572R	A>G	CAC>CGC	GAACTTGCCAACATCCTGAAATACCACATTGGTGATGAAATCCTGGTTAGC	GAACTTGCCAACATCCTGAAATACCGCATTGGTGATGAAATCCTGGTTAGC			X (position 4)	X (position 8)
	T621P	A>C	ACA>CCA	GAGCCTGACATCATGGCCACAAATGGCGTGGTCCATGTCATCA	GAGCCTGACATCATGGCCCCAAATGGCGTGGTCCATGTCATCA	X		X (position 5)	
	**Lattice Type IIIA**								
Missense	P501T	C>A	CCA>ACA	CACGATGGACCGGGTGCTGACCCCCCCAATGGGGACTGTCATGGATGTCCT	CACGATGGACCGGGTGCTGACCCCCACAATGGGGACTGTCATGGATGTCCT			X (position 1)	X (position 4)
	A546T	G>A	GCC>ACC	CACAGTCTTTGCTCCCACAAATGAAGCCTTCCGAGCCCTGCCACCAAGAGA	CACAGTCTTTGCTCCCACAAATGAAACCTTCCGAGCCCTGCCACCAAGAGA			X (position 9)	
	A620P	G>C	GCC>CCC	GCCTGTTGCCGAGCCTGACATCATGGCCACAAATGGCGTGGTCCATGTCAT	GCCTGTTGCCGAGCCTGACATCATGCCCACAAATGGCGTGGTCCATGTCAT	X		X (position 8)	
	N622K	T>A	AAT>AAA	GCCGAGCCTGACATCATGGCCACAAATGGCGTGGTCCATGTCATCACCAAT	GCCGAGCCTGACATCATGGCCACAAAAGGCGTGGTCCATGTCATCACCAAT			X (position 5)	
	N622K	T>G	AAT>AAG	GCCGAGCCTGACATCATGGCCACAAATGGCGTGGTCCATGTCATCACCAAT	GCCGAGCCTGACATCATGGCCACAAAGGGCGTGGTCCATGTCATCACCAAT	X		X (position 5)	
Small Deletion	c.1879delG		Val627SerfsX44	CATCATGGCCACAAATGGCGTGGTCCATGTCATCACCAATGTTCTGCAGCCTC	CATCATGGCCACAAATGGCGTGGTCCAT_TCATCACCAATGTTCTGCAGCCTC		X	X (position 2)	
	**Granular**								
Missense	V113I	G>A	GTT>ATT	AAACCTTTACGAGACCCTGGGAGTCGTTGGATCCACCACCACTCAGCTGTA	AAACCTTTACGAGACCCTGGGAGTCATTGGATCCACCACCACTCAGCTGTA			X (position 2)	X (position 1)
	D123H	G>C	GAC>CAC	ATCCACCACCACTCAGCTGTACACGGACCGCACGGAGAAGCTGAGGCCTGA	ATCCACCACCACTCAGCTGTACACGCACCGCACGGAGAAGCTGAGGCCTGA			X (position 7)	X (position 9)
	F547C	T>G	TTC>TGC	TTTGCTCCCACAAATGAAGCCTTCCGAGCCCTGCCACCAAGAGAACGGAG	TTTGCTCCCACAAATGAAGCCTGCCGAGCCCTGCCACCAAGAGAACGGAG			X (position 1)	
	L550P	T>C	CTG>CCG	CACAAATGAAGCCTTCCGAGCCCTGCCACCAAGAGAACGGAGCAGACTC	CACAAATGAAGCCTTCCGAGCCCCGCCACCAAGAGAACGGAGCAGACTC	X		X (position 1)	
	**Granular Type I**								
Missense	R124S	C>A	CGC>AGC	CCACCACCACTCAGCTGTACACGGACCGCACGGAGAAGCTGAGGCCTGAGAT	CCACCACCACTCAGCTGTACACGGACAGCACGGAGAAGCTGAGGCCTGAGAT			X (position 4)	
	R555W	C>T	CGG>TGG	TCCGAGCCCTGCCACCAAGAGAACGGAGCAGACTCTTGGGAGATGCCAA	TTCCGAGCCCTGCCACCAAGAGAATGGAGCAGACTCTTGGGAGATGCCAA		X	X (position 7)	
Small Deletion	c.310_311delCT		Leu103_Ser104del	cttctgctcctgcagCCCTACCACTCTCAAACCTTTACGAGACCCTGGGAG	cttctgctcctgcagCCCTACCACT_CAAACCTTTACGAGACCCTGGGAG		X	X (position 3)	
Small Indel	c.371_378delGCACGGAGinsTC		Asp123delins	CCACCACCACTCAGCTGTACACGGACCGCACGGAGAAGCTGAGGCCTGAGAT	CCACCACCACTCAGCTGTACACGGACCTCAAGCTGAGGCCTGAGATGGAGGG			X (position 7)	X (position 13)
	**Corneal Dystrophy**								
Missense	M502V	A>G	ATG>GTG	GACCGGGTGCTGACCCCCCCAATGGGGACTGTCATGGATGTCCTGAAG	GACCGGGTGCTGACCCCCCCAGTGGGGACTGTCATGGATGTCCTGAAG			X (position 1)	X (position 12)
	S516R	C>G	AGC>AGG	acattttctgtgtgtgtatctacagCATGCTGGTAGCTGCCATCCAGTCT	acattttctgtgtgtgtatctacagGATGCTGGTAGCTGCCATCCAGTCT	X	X	X (position 5)	
	L559V	T>G	TTG>GTG	CAAGAGAACGGAGCAGACTCTTGGgtaaagaccaacttaagtacacgtc	CAAGAGAACGGAGCAGACTCGTGGgtaaagaccaacttaagtacacgtc			X (position 1)	
	**Epithelial Basement Membrane**								
Missense	L509R	T>G	CTG>CGG	CCAATGGGGACTGTCATGGATGTCCTGAAGGGAGACAATCGCTTTAGgtaa	CCAATGGGGACTGTCATGGATGTCCGGAAGGGAGACAATCGCTTTAGgtaa	X		X (position 3)	
	R666S	G>C	AGG>AGC	ctttcagGCTTCCCAGAGGTCTGTGCGACTAGgtgagtctggtctgggtt	ctttcagGCTTCCCAGAGCTCTGTGCGACTAGgtgagtctggtctgggtt			X (position 4)	X (position 14)
	**Reis-Buckler**								
Missense	R124L	G>T	CGC>CTC	CCACCACCACTCAGCTGTACACGGACCGCACGGAGAAGCTGAGGCCTGAGAT	CCACCACCACTCAGCTGTACACGGACCTCACGGAGAAGCTGAGGCCTGAGAT			X (position 3)	
	L509P	T>C	CTG>CCG	CCAATGGGGACTGTCATGGATGTCCTGAAGGGAGACAATCGCTTTAG	CCAATGGGGACTGTCATGGATGTCCCGAAGGGAGACAATCGCTTTAG	X		X (position 3)	
	**Keratoconus**								
Missense	H403Q	T>G	CAT>CAG	GACAAGCCGGCCTCGGCAATCATCTCTCTGGAAGTGAGCGGTTGACCCT	GACAAGCCGGCCTCGGCAATCAGCTCTCTGGAAGTGAGCGGTTGACCCT			X (position 6)	
	G535Term	G>T	GGA>TGA	TGACGGAGACCCTCAACCGGGAAGGAGTCTACACAGTCTTTGCTCCCACA	TGACGGAGACCCTCAACCGGGAATGAGTCTACACAGTCTTTGCTCCCACA		X	X (position 5)	X (position 10)
	**Avellino/GCD II**								
Missense	R124H	G>A	CGC>CAC	CCACCACCACTCAGCTGTACACGGACCGCACGGAGAAGCTGAGGCCTGAGAT	CCACCACCACTCAGCTGTACACGGACCACACGGAGAAGCTGAGGCCTGAGAT			X (position 3)	X (position 13)
	**Combined granular-lattice type**								
Missense	M619K	T>A	ATG>AAG	GAGCCTGTTGCCGAGCCTGACATCATGGCCACAAATGGCGTGGTCCATGTC	GAGCCTGTTGCCGAGCCTGACATCAAGGCCACAAATGGCGTGGTCCATGTC			X (position 8)	
	**Granular Type II**								
Missense	R179Term	C>T	CGA>TGA	ATATGGTGGGCAGGCGAGTCCTGACTGATGAGCTGAAACACG	ATATGGTGGGCAGGTGA				
	**Map-like**								
Missense	G623R	G>C	GGC>CGC	GAGCCTGACATCATGGCCACAAATGGCGTGGTCCATGTCATCACCAATGTT	GAGCCTGACATCATGGCCACAAATCGCGTGGTCCATGTCATCACCAATGTT			X (position 4)	
	**Schnyder**								
Missense	E131D	G>T	GAG>GAT	GGACCGCACGGAGAAGCTGAGGCCTGAGATGGAGGGGCCCGGCAGCT	GGACCGCACGGAGAAGCTGATGCCTGAGATGGAGGGGCCCGGCAGCT			X (position 9)	
	**Thiel-Behnke**								
Missense	R555Q	G>A	CGG>CAG	TTCCGAGCCCTGCCACCAAGAGAACGGAGCAGACTCTTGGgtaaagaccaa	TTCCGAGCCCTGCCACCAAGAGAACAGAGCAGACTCTTGGgtaaagaccaa			X (position 8)	X (position 11)
					Total:	20/70	7 OUT OF 70	51/70	7 OUT OF 70

Supplementary Table 2: Oligo nucleotides used to clone the CRISPR/Cas9 constructs and luciferase reporter plasmids

Oligo sequence (5' to 3')	Oligo name
CACCGACTCAGCTGTACACGGACTGCA	sgR124C22ntTOP
AAACTGCAGTCCGTGTACAGCTGAGTC	sgR124C22ntBOTTOM
CACCGCTCAGCTGTACACGGACTGCA	sgR124C21ntTOP
AAACTGCAGTCCGTGTACAGCTGAGC	sgR124C21ntBOTTOM
CACCGTCAGCTGTACACGGACTGCA	sgR124C20ntTOP
AAACTGCAGTCCGTGTACAGCTGAC	sgR124C20ntBOTTOM
CACCGCAGCTGTACACGGACTGCA	sgR124C19ntTOP
AAACTGCAGTCCGTGTACAGCTGC	sgR124C19ntBOTTOM
CACCGAGCTGTACACGGACTGCA	sgR124C18ntTOP
AAACTGCAGTCCGTGTACAGCTC	sgR124C18ntBOTTOM
CACCGGCTGTACACGGACTGCA	sgR124C17ntTOP
AAACTGCAGTCCGTGTACAGCC	sgR124C17ntBOTTOM
CACCGCTGTACACGGACTGCA	sgR124C16ntTOP
AAACTGCAGTCCGTGTACAGC	sgR124C16ntBOTTOM
CACCG ACCACTCAGCTGTACACGGACCACA	sgR124H22ntTOP
AAACTGTGGTCCGTGTACAGCTGAGTGGTC	sgR124H22ntBOTTOM
CACCG CCACTCAGCTGTACACGGACCACA	sgR124H21ntTOP
AAACTGTGGTCCGTGTACAGCTGAGTGGC	sgR124H21ntBOTTOM
CACCG CACTCAGCTGTACACGGACCACA	sgR124H20ntTOP
AAACTGTGGTCCGTGTACAGCTGAGTGC	sgR124H20ntBOTTOM
CACCG ACTCAGCTGTACACGGACCACA	sgR124H19ntTOP
AAACTGTGGTCCGTGTACAGCTGAGTC	sgR124H19ntBOTTOM
CACCG CTCAGCTGTACACGGACCACA	sgR124H18ntTOP
AAACTGTGGTCCGTGTACAGCTGAGC	sgR124H18ntBOTTOM
CACCG TCAGCTGTACACGGACCACA	sgR124H17ntTOP
AAACTGTGGTCCGTGTACAGCTGAC	sgR124H17ntBOTTOM
CACCG CAGCTGTACACGGACCACA	sgR124H16ntTOP
AAACTGTGGTCCGTGTACAGCTGC	sgR124H16ntBOTTOM
CACCG ACTCAGCTGTACACGGACCTCA	sgR124L22ntTOP
AAACTGAGGTCCGTGTACAGCTGAGTC	sgR124L22ntBOTTOM
CACCG CTCAGCTGTACACGGACCTCA	sgR124L21ntTOP
AAACTGAGGTCCGTGTACAGCTGAGC	sgR124L21ntBOTTOM
CACCG TCAGCTGTACACGGACCTCA	sgR124L20ntTOP
AAACTGAGGTCCGTGTACAGCTGAC	sgR124L20ntBOTTOM
CACCG CAGCTGTACACGGACCTCA	sgR124L19ntTOP
AAACTGAGGTCCGTGTACAGCTGC	sgR124L19ntBOTTOM

CACCG AGCTGTACACGGACCTCA	sgR124L18ntTOP
AAACTGAGGTCCGTGTACAGCTC	sgR124L18ntBOTTOM
CACCG GCTGTACACGGACCTCA	sgR124L17ntTOP
AAACTGAGGTCCGTGTACAGCC	sgR124L17ntBOTTOM
CACCG CTGTACACGGACCTCA	sgR124L16ntTOP
AAACTGAGGTCCGTGTACAGC	sgR124L16ntBOTTOM
CACCG CCAAGAGTCTGCTCCATTCTCT	sgR555W22ntTOP
AAACAGAGAATGGAGCAGACTCTTGGC	sgR555W22ntBOTTOM
CACCG CAAGAGTCTGCTCCATTCTCT	sgR555W21ntTOP
AAACAGAGAATGGAGCAGACTCTTGC	sgR555W21ntBOTTOM
CACCG AAGAGTCTGCTCCATTCTCT	sgR555W20ntTOP
AAACAGAGAATGGAGCAGACTCTTC	sgR555W20ntBOTTOM
CACCG AGAGTCTGCTCCATTCTCT	sgR555W19ntTOP
AAACAGAGAATGGAGCAGACTCTC	sgR555W19ntBOTTOM
CACCG GAGTCTGCTCCATTCTCT	sgR555W18ntTOP
AAACAGAGAATGGAGCAGACTCC	sgR555W18ntBOTTOM
CACCG AGTCTGCTCCATTCTCT	sgR555W17ntTOP
AAACAGAGAATGGAGCAGACTC	sgR555W17ntBOTTOM
CACCG GTCTGCTCCATTCTCT	sgR555W16ntTOP
AAACAGAGAATGGAGCAGACC	sgR555W16ntBOTTOM
CACCG CCAAGAGTCTGCTCTGTTCTCT	sgR555Q22ntTOP
AAACAGAGAACAGAGCAGACTCTTGGC	sgR555Q22ntBOTTOM
CACCG CAAGAGTCTGCTCTGTTCTCT	sgR555Q21ntTOP
AAACAGAGAACAGAGCAGACTCTTGC	sgR555Q20ntBOTTOM
CACCG AAGAGTCTGCTCTGTTCTCT	sgR555Q20ntTOP
AAACAGAGAACAGAGCAGACTCTTC	sgR555Q21ntBOTTOM
CACCG AGAGTCTGCTCTGTTCTCT	sgR555Q19ntTOP
AAACAGAGAACAGAGCAGACTCTC	sgR555Q19ntBOTTOM
CACCG GAGTCTGCTCTGTTCTCT	sgR555Q19ntTOP
AAACAGAGAACAGAGCAGACTCC	sgR555Q19ntBOTTOM
CACCG AGTCTGCTCTGTTCTCT	sgR555Q17ntTOP
AAACAGAGAACAGAGCAGACTC	sgR555Q17ntBOTTOM
CACCG GTCTGCTCTGTTCTCT	sgR555Q16ntTOP
AAACAGAGAACAGAGCAGACC	sgR555Q16ntBOTTOM
AGATGCGGAGAAGCTGAGGCCTGAG	sgR124L AsCpf1 TOP
AAAACTCAGGCCTCAGCTTCTCCGC	sgR124L AsCpf1 BOTTOM
CACCGCCTTCCGAGCCCTGCCACCAA	sgR555W sg21 S.aureus TOP
AAACTTGGTGGCAGGGCTCGGAAGGC	sgR555W sg21 S.aureus BOTTOM
CACCGGCCTTCCGAGCCCTGCCACCAA	sgR555W sg22 S.aureus TOP
AAACTTGGTGGCAGGGCTCGGAAGGCC	sgR555W sg22 S.aureus BOTTOM
ACCACCACTCAGCTGTACACGGACCGCACGGAGAAGCTGAGGCCTGAGATG	R124 Rep WT Top
CTAGCATCTCAGGCCTCAGCTTCTCCGTGCGGTCCGTGTACAGCTGAGTGGTGGTGTAC	R124 Rep WT Bottom
ACCACCACTCAGCTGTACACGGACTGCACGGAGAAGCTGAGGCCTGAGATG	R124C Rep Mutant Top
CTAGCATCTCAGGCCTCAGCTTCTCCGTGAGGTCCGTGTACAGCTGAGTGGTGGTGTAC	R124C Rep Mutant Bottom
ACCACCACTCAGCTGTACACGGACCACACGGAGAAGCTGAGGCCTGAGATG	R124H Rep Mutant Top

CTAGCATCTCAGGCCTCAGCTTCTCCGTGTGGTCCGTGTACAGCTGAGTGGTGGTGTAC	R124H Rep Mutant Bottom
ACCACCACTCAGCTGTACACGGACCTCACGGAGAAGCTGAGGCCTGAGATG	R124L Rep Mutant Top
CTAGCATCTCAGGCCTCAGCTTCTCCGTGAGGTCCGTGTACAGCTGAGTGGTGGTGTAC	R124L Rep Mutant Bottom
TTCCGAGCCCTGCCACCAAGAGAACGGAGCAGACTCTTGGGAGATGCCAAG	R555 Rep WT Top
CTAGCTTGGCATCTCCCAAGAGTCTGCTCCGTTCTCTTGGTGGCAGGGCTCGGAAGTAC	R555 Rep WT Bottom
TTCCGAGCCCTGCCACCAAGAGAACAGAGCAGACTCTTGGGAGATGCCAAG	R555Q Rep Mutant Top
CTAGCTTGGCATCTCCCAAGAGTCTGCTCTGTTCTCTTGGTGGCAGGGCTCGGAAGTAC	R555Q Rep Mutant Bottom
CTTCCGAGCCCTGCCACCAAGAGAATGGAGCAGACTCTTGGGAGATGCCAA	R555W MUT REP TOP
CTAGTTGGCATCTCCCAAGAGTCTGCTCCATTCTCTTGGTGGCAGGGCTCGGAAGGTAC	R555W MUT REP BOTTOM

Supplementary Table 3: Off-target scores for each guide RNA utilised

		**Benchling**		**MIT Optimised CRISPR Design Tool**
		Off-target score	On-target score	Off-target score
***S.pyogenes* Cas9 NGG PAM**	**R124WT**	95	83.7	94.4
	**R124C**	62	65.4	80.1
	**R124H**	70	81.4	79.5
	**R124L**	71	47.9	82
	**R555WT**	65.6	48.1	83
	**R555Q**	46.6	0.9	31
	**R555W**	56.7	3.7	50
***S.aureus* Cas9 NNGRRT PAM**	**R555W**	30.4	79.1	Program does not look for S.aureus Cas9 PAM
**Mutant *As*Cpf1**	**R124L**	Program does not look for mutant AsCpf1 PAM		

Supplementary Figure 1: Allele-specific cleavage of L527R TGFBI mutation utilising a PAM-specific approach

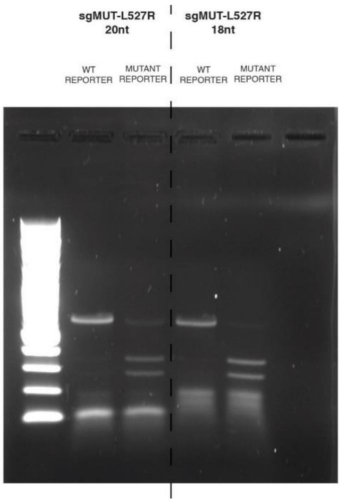

Supplementary Figure 2: Confirmation of the specificity achieved using a guide-specific system targeted to prevalent TGFBI mutations

R124C:

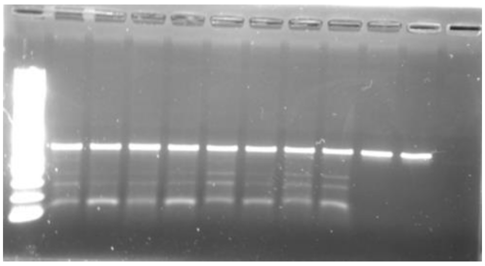

R124H:

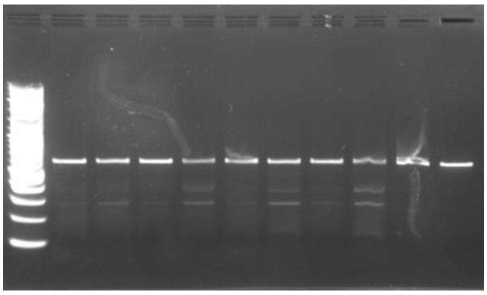

R124L:

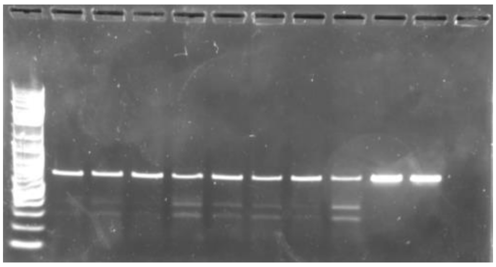

R555Q:

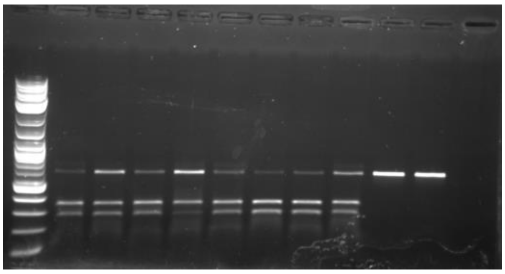

R555W:

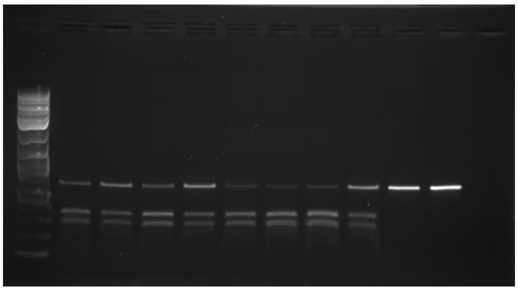

Supplement: Supplementary file 1 — Supplementary Information [file 41598_2017_16279_MOESM1_ESM.pdf]
